# Severe Thrombocytopenia Is Associated with a Genetic Variant in the Helicase Domain of *SLFN14* Gene: A Case Report

**DOI:** 10.1002/jha2.70068

**Published:** 2025-06-13

**Authors:** Kun Yang, Peixiao Fu, Jinyun Xu, Hao Jiang, Jie Yu, Chunhai Luo, Jiaowei Gu

**Affiliations:** ^1^ Department of Pediatrics Taihe Hospital Affiliated of Hubei University of Medicine Shiyan Hubei China; ^2^ Department of Dermatology Taihe Hospital Affiliated of Hubei University of Medicine Shiyan Hubei China; ^3^ Department of Urology & Andrology, Sir Run Run Shaw Hospital Zhejiang University School of Medicine Hangzhou Zhejiang China

**Keywords:** SLFN14, inherited thrombocytopenias, case report

## Abstract

Inherited thrombocytopenias (ITs) are a diverse group of hematological disorders. This study reports a novel case of severe thrombocytopenia in two male twins from nonconsanguineous parents. Whole exome sequencing (WES) identified a heterozygous genetic variant (c.1766T > C; p.L589S) in the helicase domain of the *SLFN14* gene in the twins, their mother, and maternal grandmother, while the father and maternal grandfather did not carry the genetic variant. Despite carrying the genetic variant, the mother and maternal grandmother showed no abnormal phenotypes. The twins exhibited significantly reduced platelet counts, abnormal megakaryocyte accumulation, and arrested maturation, broadening the spectrum of SLFN14‐related thrombocytopenia.

**Clinical Trial Registration**: The authors confirm that registration of a clinical trial is not necessary for this submission.

## 1 Introduction

Inherited thrombocytopenias (ITs) constitute a heterogeneous group of hematological disorders marked by congenital platelet deficiency, frequently accompanied by qualitative platelet abnormalities or systemic manifestations [1]. The advent of next‐generation sequencing, such as whole exome sequencing (WES) and whole genome sequencing (WGS), has identified >40 pathogenic loci, enabling molecular stratification of these conditions and personalized therapeutic approaches [1, 2]. Recent studies highlight the critical role of RNA metabolism regulators in megakaryocyte maturation, with SLFN14 emerging as a key player in maintaining platelet precursor homeostasis through ribosomal RNA processing [3, 4, 5].

Platelet‐type bleeding disorder 20 (BDPLT20), caused by heterozygous *SLFN14* genetic variants, represents an autosomal dominant form of genetic thrombocytopenia. *SLFN14*, a member of the *Schlafen* gene family, encodes a 912‐amino acid protein with three structural domains, including the ATPases associated with diverse cellular activities (AAA) domain involved in DNA and RNA metabolism, the SWADL region specific to SLFN proteins, and helicase domains at the C‐terminal end that play a crucial role in DNA and RNA metabolism [2]. The interaction between the N‐terminal and C‐terminal domains of SLFN14 is hypothesized to create a valley ideally suited for clamping base‐paired RNAs, facilitating subsequent RNA/rRNA cleavage, as demonstrated by a recent 3D structure of rat SLFN13, which shares high sequence homology and evolutionary conservation with SLFN14 [3, 6]. Seven heterozygous single nucleotide substitutions resulting in five distinct amino acid changes—p.K218E, p.K219N, p.K219E, p.V220D, and p.R223W—have been previously reported [3, 7–10]. These genetic variants are concentrated in a single hotspot within the AAA domain. However, to date, no clinical cases have been identified in which genetic variants in the C‐terminal helicase domain of SLFN14 are linked to severe thrombocytopenia and hemorrhagic syndrome.

## 2 Case Presentation

**FIGURE 1:**
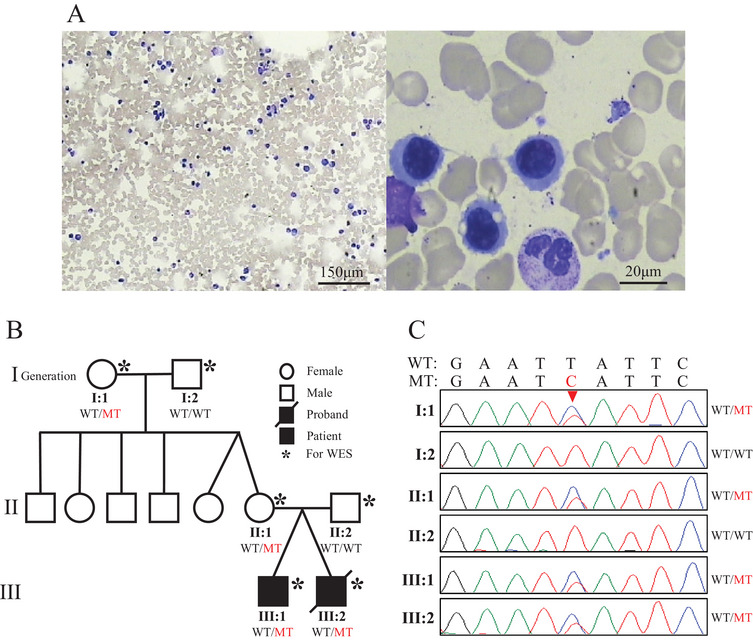
Identification of *SLFN14* genetic variants and bone marrow smear of patient. (A) Bone marrow smear of proband shows that myelodysplasia was obviously active and megakaryocyte maturation was impaired. (B) Pedigree of the patient's family. (C) Chromatograms of the *SLFN14* missense mutation (c.1766T > C) from all the available family members. WT: wild type; MT: mutation.

Here, we report a family of nonconsanguineous parents with two affected male twins with inherited thrombocytopenia. A 12‐year‐old male patient presented to our pediatric clinic with a history of severe thrombocytopenia. The patient, born prematurely 12 years ago, presented with thrombocytopenia (41 × 10^9^/L; reference 150–450 × 10^9^/L) and, in the absence of cutaneous petechiae or oral/nasal bleeding, was treated with a 2‐week course of oral prednisolone. Follow‐up assessments indicated persistent thrombocytopenia further in life (40–50 × 10^9^/L), though the patient has not experienced severe hemorrhagic symptoms during this time. The patient's twin brother also exhibits persistent thrombocytopenia without significant bleeding manifestations, while the parents and maternal grandparents have normal platelet counts and no notable bleeding symptoms. She underwent screening for TORCHES infections (toxoplasma, rubella virus, cytomegalovirus, herpes simplex virus, and syphilis) and platelet antibodies, all of which returned negative results. To further understand the hematological status of the patients, a series of laboratory tests were carried out. Routine blood tests, including complete blood counts, were conducted. The results showed that in addition to the significantly reduced platelet counts in the proband and his twin brother, other hematological parameters such as red blood cell and white blood cell counts were within the normal range. Peripheral blood smears were examined under a microscope, and the results showed that the number of platelets was significantly reduced, and the morphology of platelets, red blood cells, and white blood cells showed no obvious abnormalities other than thrombocytopenia. Bone marrow aspiration and biopsy were also performed. The bone marrow examination revealed an abnormal increase in the number of megakaryocytes, but their maturation was impaired, which is consistent with the characteristics of platelet production disorders (Figure 1A). Given the absence of significant bleeding manifestations or abnormal discomfort, and in alignment with parental preferences, no specific pharmacological treatment was initiated. Management was limited to routine platelet monitoring.

Given the persistence of thrombocytopenia and impairment of megakaryocytes maturation, a comprehensive genetic and hematological investigation was conducted. Peripheral blood samples were collected from the proband, his twin brother, parents, and grandparents, and genomic DNA was extracted for subsequent analysis. WES analysis revealed that the proband, his twin brother, mother, and maternal grandmother all carried a heterozygous genetic variant in the *SLFN14* gene (NM_001129820.2), specifically c.1766T>C (p.L589S) (Figure 1B,C). However, the father and the maternal grandfather did not have this genetic variant (Figure 1C). According to the ACMG guidelines, this variant was initially classified as Uncertain Significance (US). However, further in silico analyses strongly support its pathogenicity: Functional prediction tools, including SIFT4G, FATHMM, PROVEAN, MetaSVM, MetaLR, and MetaRNN, unanimously predicted deleterious effects; Evolutionary conservation analysis revealed a GERP++ score of 5.28 (indicating strong conservation); and pathogenicity scores, such as the CADD score of 16.03 (which exceeds the threshold of 10 for potential deleteriousness). These comprehensive in silico analyses consistently indicate a strong pathogenic effect of this variant. Based on these test results, the patient was diagnosed with Platelet—type Bleeding Disorder 20 (BDPLT20), which is caused by heterozygous *SLFN14* gene's genetic variants.

*SLFN14* genetic variant‐associated thrombocytopenia is an autosomal dominant (AD) disorder, characterized by impaired platelet function, though it has not been conclusively linked to other potentially hazardous conditions. All previously reported cases of *SLFN14* genetic variant‐associated thrombocytopenia were heterozygous and exhibited macrothrombocytopenia, with platelet counts below 150 × 10^9^/L, suggesting this as a characteristic feature of SLFN14‐related thrombocytopenia [10]. It is noteworthy that previous reports on SLFN14‐related thrombocytopenia have only described genetic variants in the N‐terminal AAA domain, although the *SLFN14* heterozygote (c.2557insC) has also been associated with atrial septal defect in a family [11]. The family reported here is the first to show a genetic variant in the helicase domain of SLFN14 associated with this clinical phenotype. Interestingly, although both the mother and maternal grandmother carry the same genetic variant, they exhibited normal platelet counts (mother: 210 × 10^9^/L; maternal grandmother: 195 × 10^9^/L) and showed no bleeding symptoms. This observation is similar to the findings reported in Family B by Fletcher et al. [2, 10].

## 3 Follow‐Up and Outcomes

The twins continue to undergo regular hematological evaluations, with platelet counts persistently ranging between 40 and 50 × 10^9^/L. No severe bleeding episodes or hemorrhagic complications have occurred since diagnosis. Management remains conservative, focusing on routine platelet monitoring and avoidance of activities with high bleeding risk (e.g., contact sports). The family was advised to promptly seek medical intervention if signs of bleeding emerge.

## 4 Discussion

In this study, we have identified a novel case of severe thrombocytopenia stemming from a genetic variant in the helicase domain of the *SLFN14* gene. This finding is of great significance as it broadens the existing spectrum of SLFN14‐related thrombocytopenia. Previous reports of SLFN14‐associated thrombocytopenia were predominantly limited to genetic variants within the N‐terminal AAA domain. Our discovery of a helicase domain genetic variant not only expands the known genetic variant landscape but also provides valuable insights into the relationship between genetic variants in different domains of the *SLFN14* gene and the resulting disease phenotypes. This emphasizes the complexity of the genotype‐phenotype correlation in SLFN14‐related disorders and suggests that genetic variants in distinct domains may disrupt different functional aspects of the SLFN14 protein, ultimately leading to thrombocytopenia through diverse molecular mechanisms.

An intriguing aspect of this case is the asymptomatic presentation in the mother and maternal grandmother, despite their carriage of the same genetic variant. This phenomenon indicates that other genetic or environmental factors likely play a role in modulating the expression of the mutant gene. Epigenetic modifications, such as DNA methylation and histone acetylation, could potentially silence or regulate the expression of the mutant *SLFN14* allele, thereby preventing the manifestation of clinical symptoms [12]. Additionally, compensatory mechanisms within their bodies might exist. For example, the upregulation of other genes involved in platelet production or function could offset the effects of the *SLFN14* genetic variant. These compensatory mechanisms could be either constitutively active or induced in response to the genetic perturbation. However, the exact nature of these epigenetic and compensatory factors remains speculative and requires further investigation.

In conclusion, this case report provides a foundation for further research into the complex relationship between *SLFN14* genetic variants and thrombocytopenia. Future studies should focus on uncovering the underlying molecular mechanisms, identifying the genetic and environmental modifiers, and translating these findings into clinical applications to improve patient care.

## 5 Patient's Perspective (from the Parents’ Perspective)

“Our sons were diagnosed with thrombocytopenia at a young age, but the absence of severe symptoms initially made it difficult to fully understand the seriousness of the condition. The identification of the genetic variant in SLFN14 provided clarity. The doctors explained that this genetic variant is rare, particularly in the helicase domain, and reassured us that close monitoring—such as avoiding rough play, using soft toothbrushes, and keeping an eye out for bruises or nosebleeds—is the best course of action for now. We are grateful for the medical team's thorough approach in ruling out other potential causes and their patience in explaining the genetic implications. For the time being, we focus on staying active within limits and hope our story helps others with this rare condition feel less isolated”.

## Author Contributions

Kun Yang and Jiaowei Gu performed the conceptualization. Kun Yang, Peixiao Fu, Jinyun Xu, Hao Jiang, and Jie Yu provided patient material. Kun Yang, Peixiao Fu, and Chunhai Luo analyzed the genetic data and hematological test results. Kun Yang, Peixiao Fu, Chunhai Luo, and Jiaowei Gu were involved in the interpretation of the results and the writing of the manuscript. All authors read and approved the final manuscript.

## Conflicts of Interest

The authors declare no conflicts of interest.

## Ethics Statement

The authors confirm that ethical approval was not required for this study.

## Patient Consent Statement

Informed consent was obtained from the patient for the publication of this case report and any related images

## Data Availability

Any aggregate data supporting the findings of this study are available from the corresponding author upon reasonable request.
